# Introductory Lecture in the Buffalo Medical College

**Published:** 1870-10

**Authors:** 


### Introductory Lecture in the Buffalo Medical College.

Prof. George Hadley will give the opening Lecture of the Course in the College Amphitheatre, Wednesday evening, November 2nd, at 7½ o'clock. The lecture will be of interest to the intelligent public, and all are cordially invited to attend.

